# Quantitative Approach for Determining Reproductive Life‐History Strategies of Parasitic Plants: A Case Study in *Balanophora*

**DOI:** 10.1002/ece3.70746

**Published:** 2025-01-11

**Authors:** Trevor Padgett, Huei‐Jiun Su, Shu‐Hui Wu, Li‐yen Huang, Yiching Lin

**Affiliations:** ^1^ International Graduate Degree Program for Biodiversity Tunghai University Taichung Taiwan; ^2^ Taiwan International Graduate Program, Biodiversity Research Center, Academia Sinica Taipei Taiwan; ^3^ Department of Earth and Life Sciences University of Taipei Taipei Taiwan; ^4^ Forest Ecology Division, Taiwan Forestry Research Institute Ministry of Agriculture Taipei Taiwan; ^5^ Hengchun Research Center, Taiwan Forestry Research Institute Ministry of Agriculture Hengchun Taiwan; ^6^ Department of Life Science Tunghai University Taichung City Taiwan

**Keywords:** Balanophoraceae, holoparasite, iteroparity, perennial, semelparity, traits

## Abstract

Parasitic plants are a diverse and unique polyphyletic assemblage of flowering plants that survive by obtaining resources via direct vascular connections to a host plant. Ecologically important in their native ecosystems, these typically cryptic plants remain understudied and fundamental knowledge of the biology, ecology, and evolution of most species is lacking. This gap limits our understanding of ecosystems and conservation management. We established a multistep protocol to conduct the first investigation of the reproductive life history of root parasite genus *Balanophora*, testing the hypotheses of perenniality, cryptic perenniality, and plasticity across five geographically isolated populations in Taiwan. A review of 123 *Balanophora* publications found contradictory determinations, including no determination (87%), perennial (9%), annual (1%), biennial (1%), or a combination (2%). No primary study investigated the question, and no determination was accompanied by reference. Between 2021 and 2024, we tested a hypothesis of perenniality (109 individuals, 135 patches) and cryptic perenniality (73 host samples), monitored population dynamics (whole population), and potential for endophytic/dormant haustorial tissue (101 roots) across five isolated populations of *Balanophora fungosa* ssp. *fungosa* in Taiwan. Our results support semelparous annuality. After reproduction, individuals senesce and die, and the following year's population is recruited from newly germinated individuals which together develop in size and number during a vegetative growth period, undergo reproduction, and then themselves senesce and die. Each cycle is completed within a 12‐month period. Synthesis: Our study provides the first quantitative determination of a semelparous annual reproductive life‐history strategy for any species of *Balanophora*. This determination is important in our progress toward better understanding the species—and parasitic plants in general—as well as ecological roles within ecosystems and conservation management. Our study further provides a template for future work to expand life‐history strategy determination across cryptic root parasitic plants.

## Graphical abstract

Life‐history strategies of parasitic plants are infrequently investigated and remain a gap in our ecological and evolutionary understanding of these important but understudied plants and the ecosystems in which they grow. To address this, we developed a robust literature review and field study protocol to test a hypothesis of perenniality, using *Balanophora fungosa* ssp. *fungosa* as a case study. Our results provide clear evidence of semelparous annuality, and our methodology is a replicable protocol that we encourage others to apply to parasitic plants to expand our fundamental understanding of this unique heterotrophic cohort of plants.

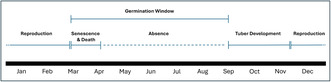

## 1 Introduction

Parasitic plants are a unique assemblage of flowering plants that have lost the ability to survive independently as autotrophs and instead survive by obtaining necessary resources from a host plant via vascular connection(s) made by a specialized organ called a haustorium (Heide‐Jorgenson 2008; Kokla and Melnyk 2018; Kujit 1969; Press and Graves 1995; Teixeira‐Costa 2021; Yoshida et al. 2016). The parasitic habit is polyphyletic, having evolved 12 independent times, is diverse, comprising more than 4750 known species (ca. 1% of flowering plant species), and has a global distribution (Nickrent 2020; Watling and Press 2001; Westwood et al. 2010). This diverse assemblage includes stem, root, and endophytic specialists that can be partially (hemiparasitic) or fully (holoparasitic) reliant on their hosts (Nickrent 2020; Těšitel, Plavcová, and Cameron 2010).

Although they survive by removing resources from their hosts and provide little in return (Clarke et al. 2019; Delavault et al. 2017; Irving and Cameron 2009), parasitic plants are increasingly found to play keystone—sometimes positive—roles in their native ecosystem. Through both direct and indirect interactions with biotic and abiotic ecosystem components, parasitic plants have variously been shown to promote ecosystem diversity; provide otherwise lacking foliar, fruit, and nectar resources during times of scarcity; increase nutrient cycling and soil nutrient load; increase pollination success of nearby plants; and provide important niche space for both avian and invertebrate communities, with influences disproportionate to their biomass or population (March and Watson 2007; Hautier et al. 2010; Pierce and Olge 2017; Press and Phoenix 2005; Suetsugu 2019, 2020; Suetsugu and Hashiwaki 2023; Suetsugu and Hisamatsu 2020; Quested 2008; Těšitel et al. 2021; Watson 2009; Watson, McLellan, and Fontúrbel 2022; Watson and Herring 2012; Wood et al. 2012).

However, outside of a few economically important crop pests, parasitic plants comprise some of the least studied of the flowering plants (Irving and Cameron 2009; Nickrent 2002; Parker and Riches 1993; Westwood et al. 2010). While understudied taxa on the whole present a substantive and limiting gap in our understanding of ecosystem dynamics and evolutionary patterns (Dorey, Lendemer, and Naczi 2018; Dos Santos et al. 2020; Haelewaters et al. 2024; Ritter et al. 2019), including the lack of basic ecological and natural history data (Nanglu et al. 2023; Schmidly 2005), the unique and diverse ecology and evolution of parasitism make the understudied cohort of parasitic plants a substantial gap with singular potential. Their unique habits and host relationships, combined with an observed disproportionate ecosystem influence that is putatively shared across the speciose, diverse, and phylogenetically dispersed assemblage, make parasitic plants a compelling group of plants to study. Their nascent potential provides a valuable opportunity to investigate these species themselves, but importantly also novel and pressing ecological and evolutionary questions (Armstrong et al. 2023). In providing the first quantitative investigation of reproductive life‐history strategies of the understudied obligate parasitic plant *Balanophora fungosa* ssp. *fungosa*, we provide resolution on a currently unknown component of its natural history. In doing so, we aim to fill a currently limiting gap in our understanding of the species and its ecosystem interactions, while also providing requisite data on a key life‐history strategy necessary to properly interpret ecological, genetic, and population data.

Life‐history strategies are adaptive responses to an environment, wherein resources are differentially allocated in a somewhat zero‐sum “hedge‐betting” game to specific phenotypes or physiologies that increase survival and/or reproductive success (Adler et al. 2013; Bufford and Daehler 2011; Lundgren and Des Marais 2020; Salguero‐Gómez et al. 2016). Life‐history strategies in plants encompass a wide range of morphological and physiological traits and their processes over time (Stearns 1977; Alder et al. 2013) and are known to show plasticity among (Boyko et al. 2023; Felmy et al. 2022; Kelly et al. 2021; Reynolds et al. 2017) and within (Childs, Metcalf, and Rees 2010; Simons 2011) populations. Determining population‐specific strategies is, therefore, fundamental and necessary to properly understand and interpret the biology, ecology, and evolution, as well as the conservation management, of a given species (Salguero‐Gómez et al. 2016; Stott et al. 2024).

Reproductive life‐history strategies can be broadly grouped into polycarpic, reproducing multiple times throughout a multiyear lifespan (i.e., iteroparity; *perennials*), or monocarpic, reproducing only once during a shortened, usually 1 year, lifespan (i.e., semelparity; *annuals*) (Albani and Coupland 2010; Friedman 2020; Hjertaas et al. 2023; Hughes 2017; Symonides 1988). These strategies can be conceptualized as evolutionary bet‐hedges, with perennial species hedging on long‐term survival of the parent over seed production per reproductive event, whereas annual species hedge on single mass seed production events over the long‐term survival of the parent (Adler et al. 2013; Friedman 2020; Hjertaas et al. 2023; Tilman and Wedin 1991; Vico et al. 2016).

The reproductive strategy a given species has evolved is not predicted by either phylogeny or environment. With thousands of independent evolutions, lineages exhibiting derived annuality from ancestral perenniality are highly phylogenetically dispersed and numerous unique genetic pathways to annuality have been identified (Grillo et al. 2009; Heidel et al. 2016; Hjertaas et al. 2023; Rehman, Bahadur, and Xia 2023). With the added complexity of the potential for reversions from derived annuality back to perenniality (e.g., Monroe et al. 2019) and conspecific plasticity (Hall and Willis 2005; Van Kleunen 2007), phylogenetic lineage is not an accurate predictor (Böhle, Hilger, and Martin 1996; Heidel et al. 2016; Van Kleunen 2007). Similarly, although our understanding of broad‐scale evolutionary patterns that promote and preferentially select for either strategy is well known (Gould, Chen, and Lowry 2017; Lowry et al. 2019; Lundgren and Des Marais 2020; Poppenwimer, Mayrose, and DeMalach 2023; Stearns 1992), there exists great diversity of strategies within a given ecosystems. The determination of site‐specific drivers for individual species remains incomplete (Friedman 2020; Friedman, Middleton, and Rubin 2019), and as a result, site‐specific determinations of a given species' strategy are important.

Perenniality and annuality are both observed in parasitic plants (Press and Phoenix 2005) but are rarely reported. When designations are made, they are often based on short‐term observation rather than quantitative investigation (e.g., *Cuscuta epithymum* ; Dean 1954; Meulebrouck et al. 2009). These short‐term observational data can be misleading, causing important errors in interpretation. For example, cryptic perenniality—where a perennial species appears to be annual—could occur as the result of numerous situations: If a perennial parasite parasitizes an annual host, if hosts undergo significant population losses (e.g., Schneeweiss 2007), if a perennial parasite undergoes vegetative dormancy (Dean 1954; Meulebrouck et al. 2009), or if a perennial parasite is endophytic, requiring microscopy properly determines the longevity of the parasitic vegetative tissue within a host (Teixeira‐Costa 2021). The existing gap in the body of knowledge of reproductive life‐history strategies of parasitic plants limits our understanding of these understudied species and the ecosystems within which they exist.

To address this knowledge gap, we built a protocol to test the hypothesis of perenniality using *Balanophora fungosa* ssp. *fungosa* (Balanophoraceae) in Taiwan as a case study. *B. fungosa* ssp. *fungosa* is an achlorophyllous herbaceous obligate root parasite that in Taiwan occurs in five geographically isolated populations, each with unique habitat conditions and forest assemblages (i.e., potential hosts). The plant consists of a belowground tuber affixed to a host root, from which during reproductive seasons grow emergent monoecious inflorescence‐like structures (Barkman, Emoi, and Repin 2003; Eberwein, Nickrent, and Weber 2009; Hansen 1972; Hsiao, Mauseth, and Peng 1995; Shivamurthy, Arekal, and Swamy 1981; Shivamurthy, Swamy, and Arekal 1981). The genus is cryptic; following reproduction the inflorescences dehisce, leaving behind the belowground tubers where they could potentially remain alive but inactive for periods of time (Shefferson 2009; Tamm 1948; Wilbur and Rudolf 2006). It currently remains unknown if *Balanophora* tubers remain living for multiple years and undergo multiple reproductive events (i.e., perennial), or senesce and die after a single reproductive event (i.e., annual). Clarifying this life‐history strategy is fundamental to our understanding of the species and its conservation management.

To answer this question, we first conducted a literature review of the genus *Balanophora* to determine the range of published strategies and the strength of their evidence. Using tubers (root–stem–leaf analog in *Balanophora*) to represent the individual, we then tested six hypotheses of perenniality. We first hypothesized that if *B. fungosa* ssp. *fungosa* is a perennial species, then:

- A substantial portion of tubers will survive post‐reproduction
- Tubers will persist in the respective forests between reproductive seasons
- Yearly tuber populations will remain relatively stable
- Tuber volumes will remain relatively constant or increase from previous years' volumes

We then hypothesized that if *B. fungosa* ssp. *fungosa* is a cryptic perennial, wherein a perennial species exhibits annual characteristics, then:

- The main hosts at each site will be annual species
- Tubers will be either endophytic or dormant

We present this structure of testing the putatively ancestral perennial strategy as a method that can be applied across cryptic root parasitic flowering plants. Accurate determination of reproductive life‐history strategies not only provides an opportunity to better our understanding of their biology and ecological roles but also further increases our accuracy in analyzing and interpreting ecological and genetic data, which contribute to their conservation.

## 2 Methods

### 2.1 Study Sites

**FIGURE 1:**
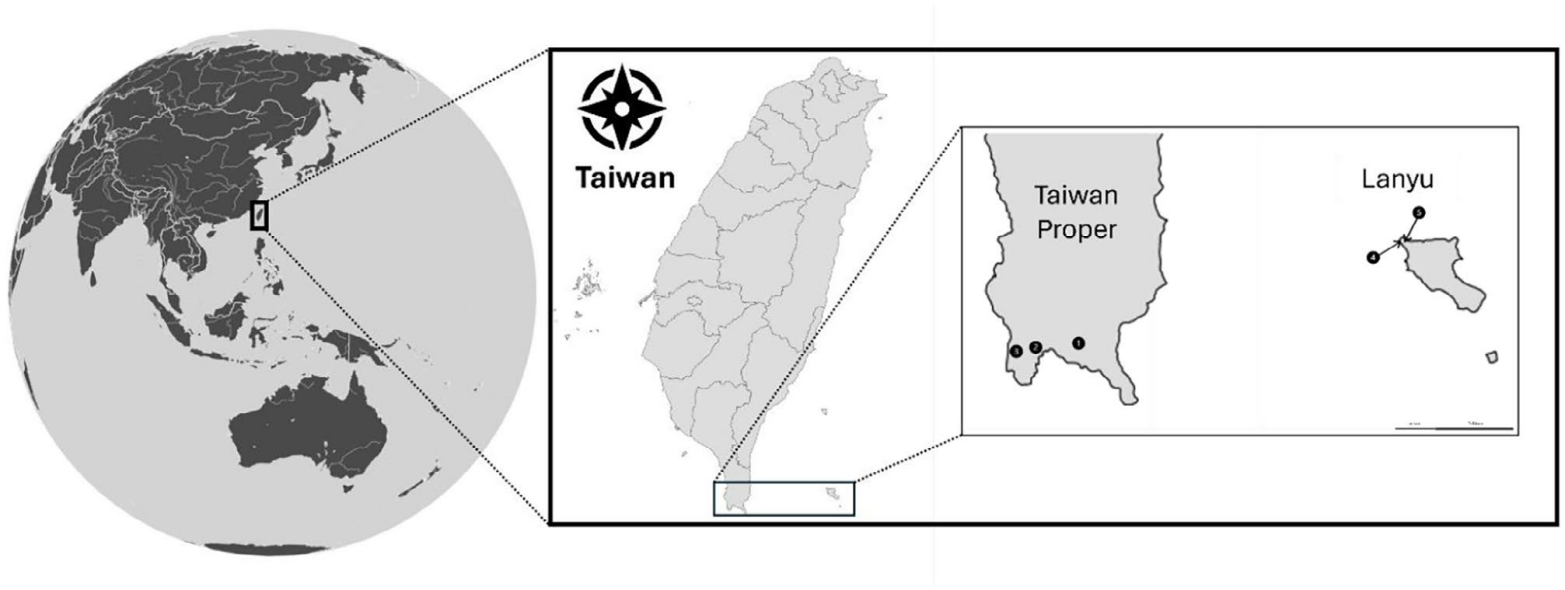
Geographical distribution of the five Taiwan populations of *B. fungosa* ssp. *fungosa*. Three populations occur on limestone substrate (uplifted coral reef) on Taiwan proper, two occur on volcanic andesite substrate on the island of Lanyu. All populations are geographically isolated (Lanyu—Kenting distance ca. 80 km; Taiwan proper populations separated 2–10 km).

This study was conducted across five geographically isolated populations in southern Taiwan, comprising the entire known Taiwan population of *B. fungosa* ssp. *fungosa* (Figure 1). All sites are forested and composed of tropical evergreen tree species that are floristically closer to Southeast Asian flora than that of the rest of Taiwan (Chao et al. 2010; Li et al. 2013; Wu et al. 2011) and strongly affected by seasonal monsoons. Three populations occur on Taiwan Proper, and two on the offshore volcanic island of Lanyu. Taiwan Proper and Lanyu populations are separated by > 80 km.

All three Taiwan proper populations are underlain by uplifted Pleistocene coral reef limestone and experience a distinct dry season (November–March). The Kenting Forest Dynamics Plot (hereafter “Kenting”; 21°57′ N, 120°48′ E; ca 250 m a.s.l.) is a 10‐ha long‐term forest dynamics plot established in 1996 by the Taiwan Forest Research Institute and is dominated by *Diospyros maritima* (Ebenaceae; > 50% of stems) (Wu et al. 2011). Plot perimeter surveys (*n* = 2; 20 m intervals) and linear transects perpendicular to the plot boundaries (*n* = 4; 200 m each) were conducted in 2022–2023 and 2023–2024 to verify that the *B. fungosa* ssp. *fungosa* population is mostly restricted to the 10‐ha plot (ca. 50 individuals in five patches observed in non‐plot surveys). Guanshan Valley (hereafter “Guanshan”; 21°58′, 120°43′; ca 60 m a.s.l.) is a ca. 2‐ha relict primary forest surrounded by agricultural land and secondary forest dominated by invasive *Leucaena leucocephela* (Fabaceae). Dominant species include *Diospyros philippensis* (Ebenaceae), *Kleinhovia hospita* (Malvaceae), and *Calophyllum inophyllum* (Calophyllaceae). Houbihu (21°55′ N, 120°44′ E; ca. 5 m a.s.l.) is a ca. 1‐ha monodominant *Hibiscus taiwanensis* (Malvaceae; > 90% of stems) forest located ca. 20 m from the coastline. The two Lanyu populations are geographically proximate (ca. 120 m), but are interrupted by discontinuous forest cover and a ca. 300‐m deep vertical‐walled valley. Both sites are underlain by andesite‐derived soils, experience consistent year‐round precipitation, and have diverse tropical tree assemblages including *Gomphandra luzoniensis* (Stemonuraceae), *Endiandra* spp. (Lauraceae), *Ardisia* spp. (Primulaceae), *Excoecaria kawakamii* (Euphorbiaceae), *Leea philippensis* (Vitaceae), *Syzygium* spp. (Myrtaceae), and *Dysoxylum* spp. (Meliaceae). Lanyu‐Lighthouse (hereafter ‘Lanyu‐1’; 22°4′ N, 121°30′ E; ca. 300 m a.s.l.) is a ca. 5‐ha forest fragment bordered by steep cliffs and a lighthouse. Lanyu‐Xiao Tian Chih (hereafter “Lanyu‐2”; 22°4′ N, 121°30′ E; ca. 250 m a.s.l.) occurs on the slopes (ca. 40° slope angle) of an extinct volcano and an adjacent inland river valley.

### 2.2 Focal Species & Growth Habit

**FIGURE 2:**
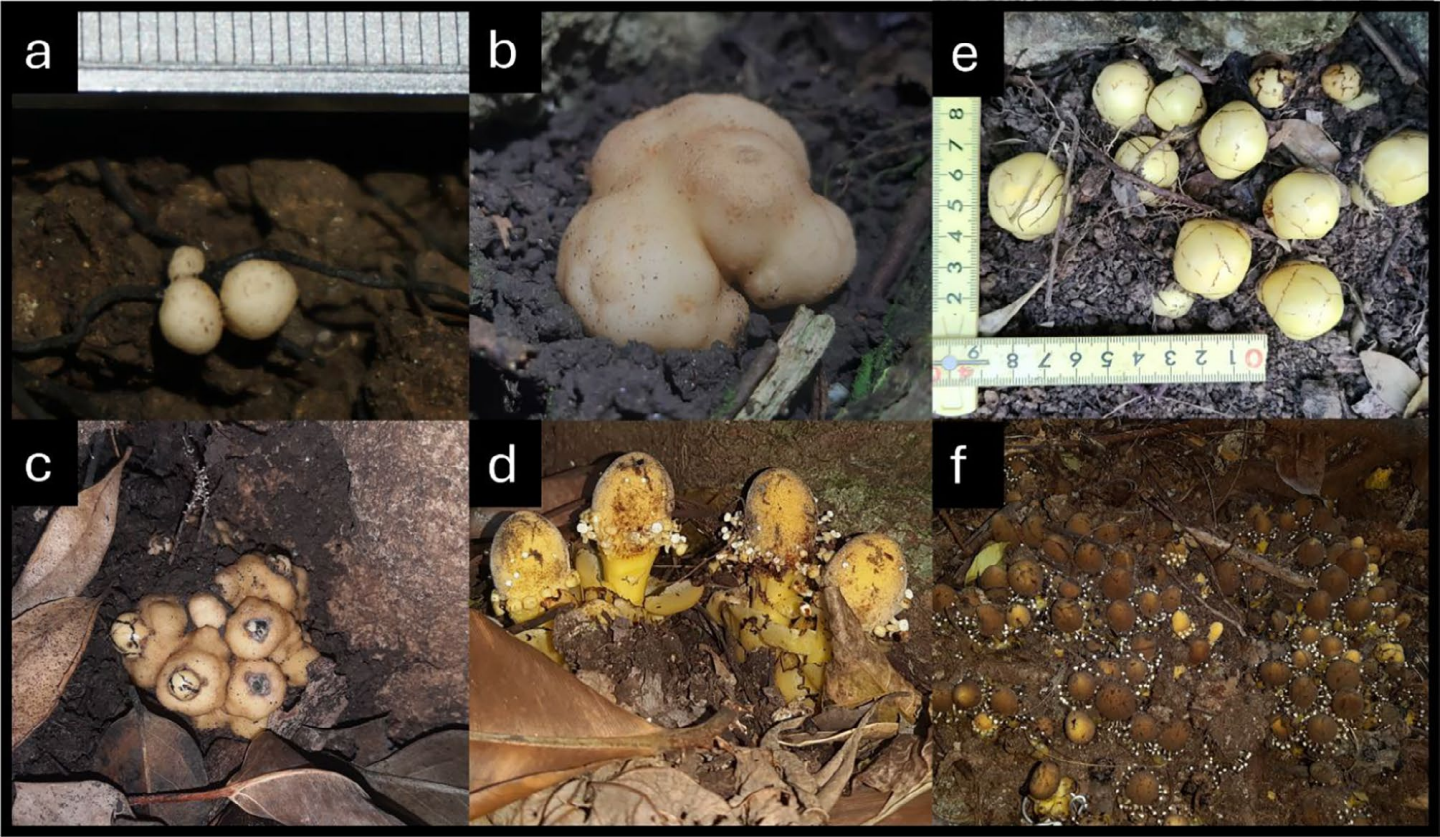
Tuber development following germination and haustorial connection are first observed as < 1 mm proto‐tubers (a); vegetative growth of the tuber continues (b); inflorescence buds emerge and develop, one to a few per tuber (c); vegetative growth ceases and monoecious inflorescence‐analogous structures develop (d). Tubers spatially arranged in patches, from ca. 10 (e; inflorescence‐analogous buds shown) to > 100 (f; mature seed‐bearing individuals shown).

*B. fungosa* ssp. *fungosa* is morphologically highly reduced compared to other flowering plants, consisting of only a usually subterranean tuber and an emergent inflorescence‐analogous structure. Tuber growth initiates with germination of the ca. 0.3 mm “dust seeds” (Hansen 1980; Shivamurthy, Arekal, and Swamy 1981; Suetsugu 2020) which first anchor to a host root and then produce a haustorium that establishes vascular connection between the pair (Shivamurthy, Arekal, and Swamy 1981). Over time this proto‐tuber develops in size until reaching reproductive maturity (ca. 2–4 cm diameter), at which point vegetative development ceases and monoecious inflorescence‐analogous structures emerge (Eberwein, Nickrent, and Weber 2009). Following reproductive maturity, inflorescences senesce and dehisce from the tuber. The tuber constitutes the vegetative “body” of the plant and is used as the unit of measure for developmental patterns and tests for perenniality. In all five Taiwan populations, tubers are spatially arranged into patches of ca. 10 to > 100. Each patch is isolated from nearest patch by up to 40 m (Figure 2). There is no other species of *Balanophora* sympatric in any population, negating potential misidentification of tuber species.

### 2.3 Literature Review

We conducted a comprehensive literature review of *Balanophora* publications to identify reported reproductive life‐history strategies. We included all ecological‐based publications of the genus obtained from Google Scholar, Google Search, JSTOR, online herbaria and floras, and the plant collections and library of the Herbarium of Academia Sinica, Taiwan. Pharmacological or medicinal publications were omitted. For each publication, we asked: (i) is a strategy reported?, (ii) is the reported strategy supported by data or reference(s)?, and (iii) do the provided data or reference(s) support the reported strategy?

### 2.4 Post‐Flowering Tuber Health

We monitored the post‐reproduction health of a subset of tubers in four populations for 2 years (*n* = 109 tubers) in 2023 (*n* = 14 tubers; two Taiwan Proper sites, two Lanyu sites) and 2024 (*n* = 95 tubers; one Taiwan Proper site, two Lanyu sites). Health was determined by colour, texture/physical integrity, and interior condition of tubers before and 1–2 months after reproduction. Healthy living *Balanophora* tubers are yellow (Taiwan Proper) or dull pink (Lanyu) with a firm structure, resembling that of a fresh potato. Tuber colour was tested by careful excavation by hand or trowel and washing to remove adhering soil. Tuber integrity was tested by applying pressure by pressing between the thumb and forefinger and observing resistance to deformation. Tuber interior condition was tested by either cutting (tubers that did not deform with pressure) or further squeezing and opening by hand (tubers that did deform with pressure).

### 2.5 Persistence of Tubers

Each population was systematically surveyed during the 2021–2022, 2022–2023, and 2023–2024 reproductive seasons (December–March), during which every *B. fungosa* ssp. *fungosa* patch was geolocated and tagged with a unique identifying patch number. In June or September, a preselected subset of these patches in Kenting (*n* = 108; ca. 50% of total patches), Guanshan (*n* = 3; 75%), Houbihu (*n* = 11 patches; 80%), Lanyu‐1 (all patches), and Lanyu‐2 (all patches) were surveyed and the number of tubers persisting in each patch was recorded. The surveyed patches accounted for ca. 2100 individual tubers present during the reproductive season, representing ca. 35% of total tuber populations on Taiwan Proper and 100% on Lanyu. We established a purposely low threshold of 20% persistence to support perenniality.

### 2.6 Patch Demography and Tuber Volume

We conducted nearly biweekly surveys of the volume and number of tubers in a subset of patches in Kenting (2023, *n* = 21; 2024, *n* = 41) and Guanshan (2023, *n* = 1) during the vegetative growth period (August—December). Our sampling coverage accounted for ca. 6%, 25%, and 35% of total patches, respectively. Tuber volume was measured as an ellipsoid, with three axial measurements: *v* = 4/3*axis‐A/2*axis‐B/2*axis‐C/2.

### 2.7 Population Fluctuation

Population surveys (number of patches and tubers) were conducted during the reproductive season (December–March). Qualitative observations (comparative assessment of population sizes; 2018–2021) and systematic whole‐plot surveys (10‐m interval transect surveys; 2021–2024) were conducted in Kenting. In the remaining four populations, a 5‐m radius around every tree was surveyed (2022–2023 and 2023–2024). Each year all patches were geolocated and tagged, and number of tubers per patch was recorded.

### 2.8 Reproductive Life History of Hosts

Host ranges were identified in each population by either molecular barcode, root tracing, or proximity (monodominant forest only). Molecular identification (Kenting *n* = 48; Guanshan *n* = 2; Lanyu‐1 *n* = 17; Lanyu‐2 *n* = 4) was conducted by sampling a root section (ca. 1 cm) on the non‐tree side of the tuber. Root tissue was dry stored in silica gel in the field and then transferred to a −20°C freezer. Before extraction, roots were cleaned with ddH_2_O and a 1‐min 75% EtOH bath and then air‐dried. DNA was extracted using the CTAB workflow (Doyle and Doyle 1987) with DNeasy Plant Min Kit (Qiagen) cleanup. *trn*‐L and *trn*‐F primers were used for PCR (Taberlet et al. 1991). PCR product was sent to Genomics (Taipei, Taiwan) for Sanger sequencing. Raw reads were edited and trimmed using Chromas (version 2.6.6) and alignment and consensus sequences formed using BioEdit (version 7.7.1). Each consensus sequence was compared to the NCBI library using nucleotide BLAST searches. Root tracing (Guanshan *n* = 2; Lanyu‐1 *n* = 4; Lanyu‐2 *n* = 6) was conducted only if an infected root could be followed back to its parent tree. Proximity (Houbihu *n* = 8) was only used in the monodominant forest (*Hibiscus taiwanensis* = > 95% stems).

### 2.9 Endophytic or Dormant Haustorial Parasite

**FIGURE 3:**
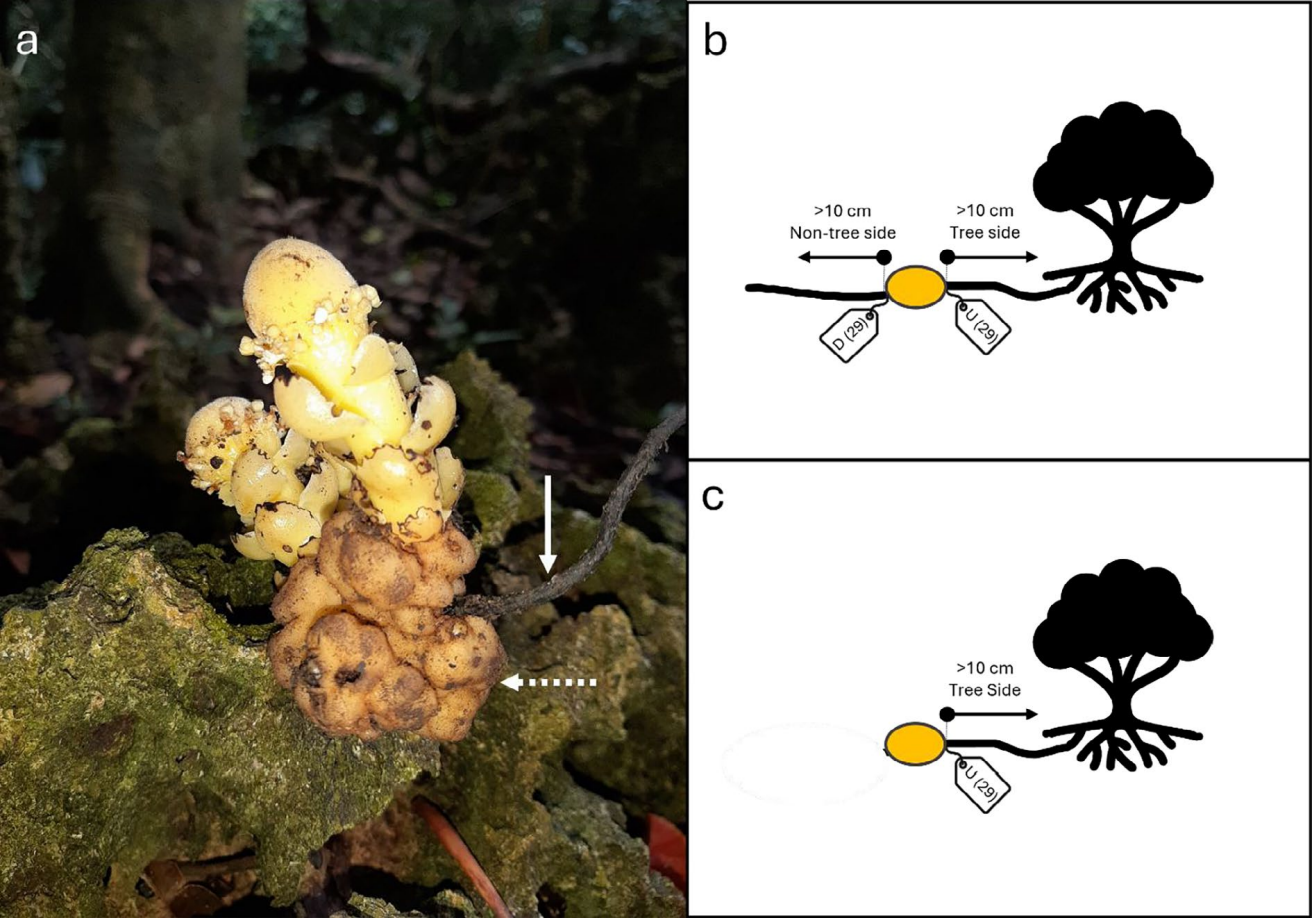
To identify an endophytic or dormant habit, tags were affixed at the tuber (dashed arrow) and root (solid arrow) interface (a) on both the tree and non‐tree side of each tuber. Root sections between tags as well as > 10 cm on both the non‐tree and tree side of the root section were checked for new tuber growth (b). In the cases when the non‐tree side of the root section was not present, the infected root section and > 10 cm upstream were surveyed (c).

We tagged a subset of host roots immediately on the tree side and non‐tree side of the tuber (*n* = 51 total; Kenting *n* = 35, Guanshan Valley *n* = 2, Houbihu *n* = 5, Lanyu‐1 *n* = 3, and Lanyu‐2 *n* = 6). Tagged roots were monitored a minimum of 10 cm up‐ and downstream of tagged root sections during two subsequent reproductive seasons (Figure 3). All tubers that re‐grew within the ca. 20 cm section of root were considered potential endophytes or dormant tubers.

## 3 Results

### 3.1 Literature Review

Our literature review found no primary investigation into the reproductive life‐history strategy of *Balanophora* (**S1**). Publications (*n* = 123) were from 1844 to 2024 in English, German, Vietnamese, Japanese, Indonesian Bahasa, and Latin; translations were conducted by either native speakers or online translation services where necessary. Roughly half (ca. 48%) of the publications were between 1844 and 2000 and half (ca. 52%) between 2001 and 2023.

Most of the publications did not report a reproductive life‐history strategy (*n* = 106; 87%). Reported strategies were perennial (*n* = 12; 9.8%), annual (*n* = 1; ca. 0.8%), biennial/multi‐year (*n* = 1; ca. 0.8%), and either perennial or annual (*n* = 2; ca. 1.6%). No publication that reported a strategy provided primary data nor valid reference.

### 3.2 Tuber Health

**FIGURE 4:**
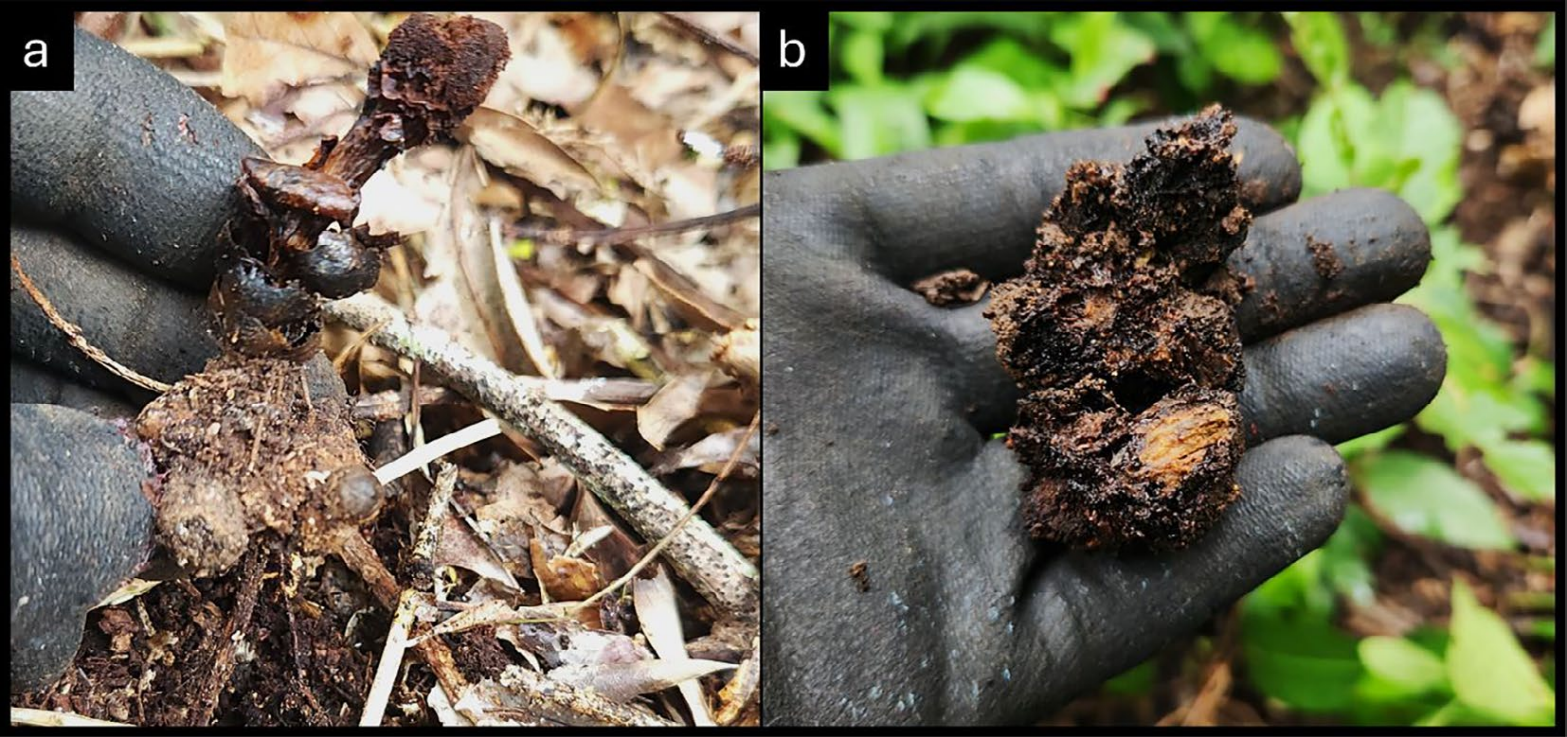
Post reproduction tubers begin to discolour (a) and show the signs of senescence then death, eventually becoming soft and deformable with slight pressure (b).

All surveyed tubers (100%) showed signs of senescence following reproduction (Figure 4). All tubers exhibited discolouration (2023 *n* = 14, 2024 *n* = 95) and most had already begun deterioration (*n* = 104), being easily deformed between thumb and forefinger. Those that maintained their structure after squeezing (*n* = 5) were dissected with a knife in the field and showed signs of interior deterioration and discolouration.

### 3.3 Persistence of Tubers

**FIGURE 5:**
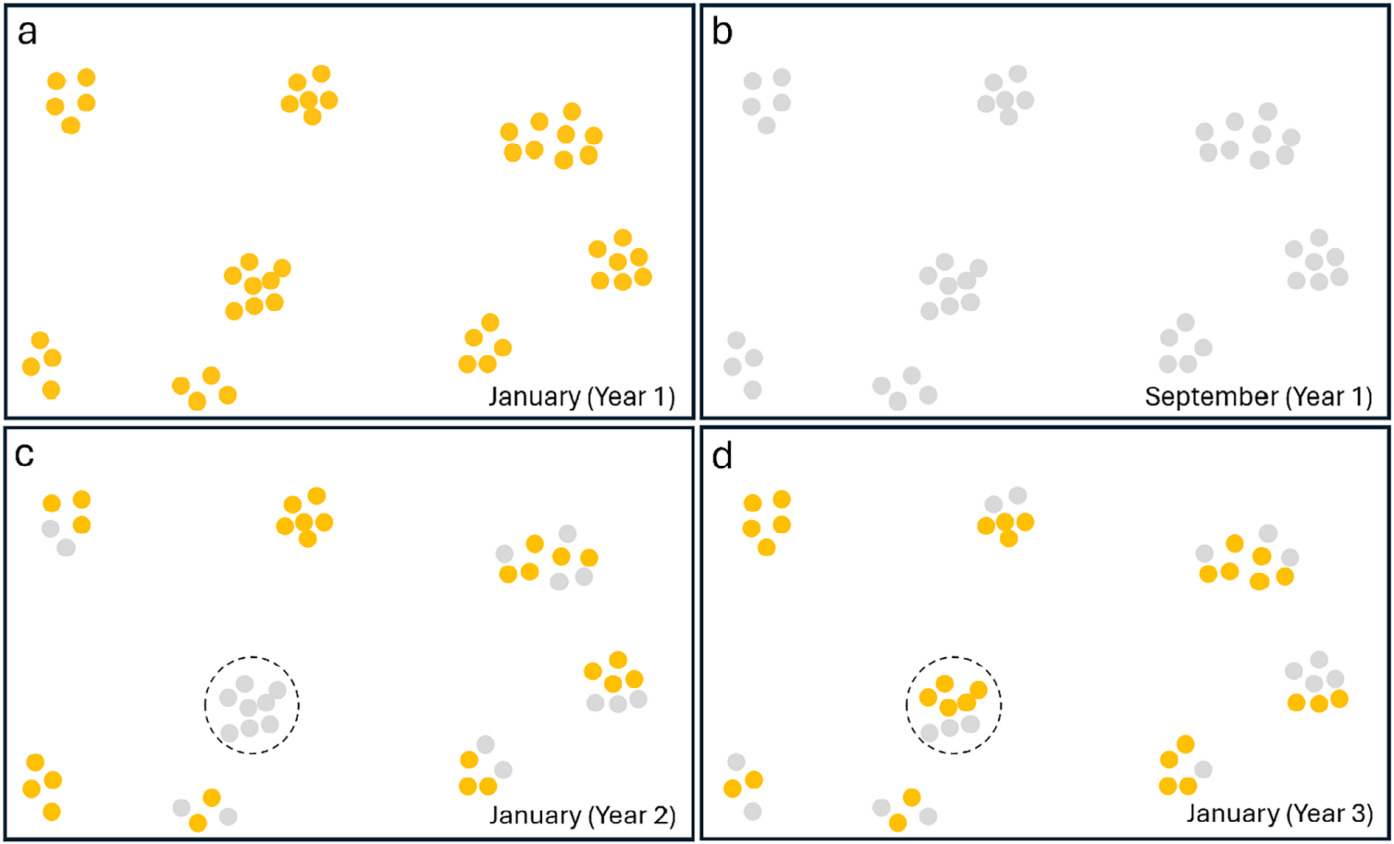
Conceptual model representing tuber persistence and patch dynamics across years. Annual whole‐population surveys geolocated and marked patches at each site (a), finding an absence of patches (no tubers) between flowering seasons (b) but year‐to‐year fidelity in patch location during the flowering seasons (c, d). Yearly differences in patch population sizes (orange and gray dots representing population changes) and the presence of “zombie” patches (present in the first and third year but missing in the second were observed (dashed circle)).

No tubers (0%) in monitored patches were found to remain present in 2021 (June), 2022 (June) or 2023 (June and September) (Figure 5). During surveys in Kenting, patches occurring in caves or cave‐like crevices were observed to have numerous dried/blackened tubers present. Protection from precipitation in these habitats promoted desiccation and prevented rotting. These tubers were additionally checked for vitality, finding that none were living, all highly desiccated and easily crumbling with a gentle touch.

### 3.4 Patch Demography and Tuber Volume

**FIGURE 6:**
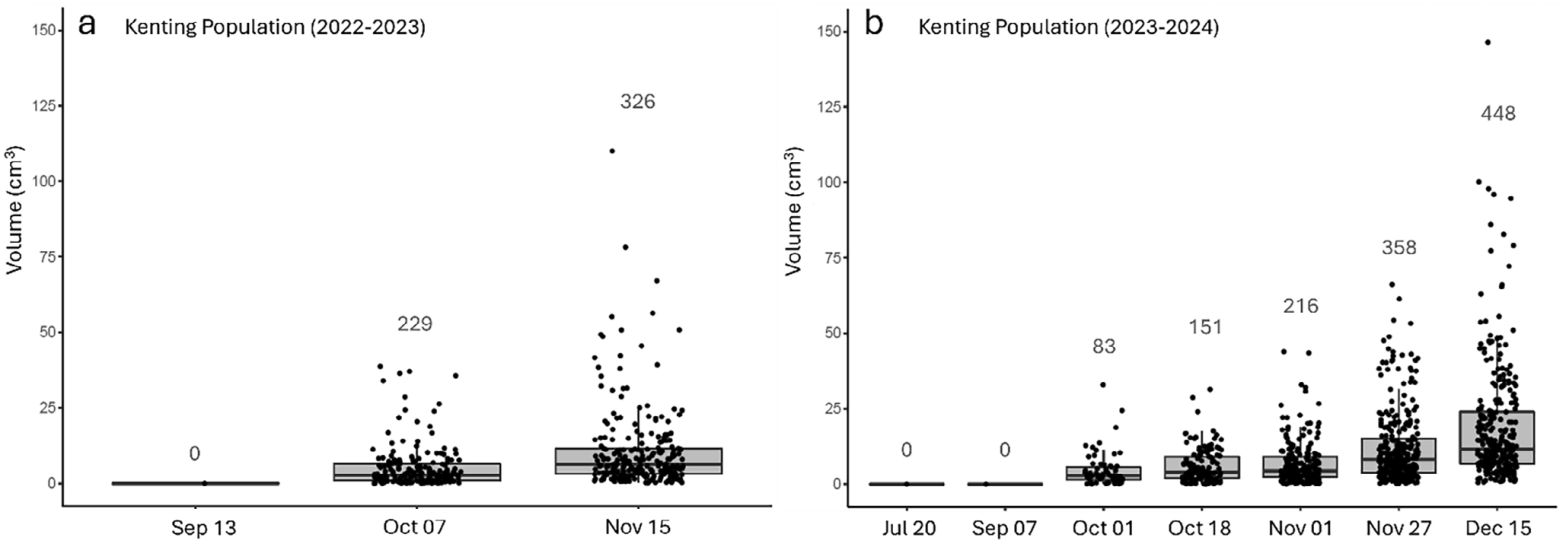
Tuber volume (box plots and solid dots) and population (numbers) of tubers in surveyed patches at each time point for a subset of patches in Kenting during the 2022–2023 (a) and 2023–2024 (b) reproductive seasons. Tuber volume and number in both years increased over the course of vegetative growth season from zero (i.e., tubers absent) until the onset of peak flowering when measurements were ceased.

Tuber volumes and number per patch in Kenting (2022–2023, *n* = 21 patches; 2023–2024, *n* = 41 patches) increase progressively from zero (Jul/Sep) until reproductive season when measurement was ceased (Dec) (Figure 6). In 2023–2024, the entire population of Guanshan was absent (accounting for four patches and ca. 150 individuals) and therefore excluded from the survey.

Tubers (< 1 mm diameter) first became noticeable in September, and increase in volume and number per patch over months. However, new (< 1 mm) tubers appear throughout the vegetative growing period, maintaining a low average patch volume but still showing increased tuber numbers. Surveys also revealed tuber deaths during vegetative growing period; dead tubers would either rot or desiccate and remain affixed to host root. To represent the total reproductive effort per patch, we report tuber volume as the volume of currently living tubers and tuber number as the total living plus dead tubers. The death of tubers also artificially lowers tuber volume data at each time point.

### 3.5 Population Fluctuation

Populations fluctuated substantially across years and were not consistent in either trend nor direction of change across sites or years, but within site directions of change were consistent (Table 1). Kenting remained the largest population over the surveyed period and underwent substantial year‐to‐year changes in both qualitative (observation, *n* = 2) and quantitative (surveyed, *n* = 3) years. Based on the results of the quantitative survey data, observations (qualitative) from previous years suggest population sizes fluctuated from large (2018–2019; ca. 3000) to small (2019–2020; ca. < 1000), to medium (2020–2021; ca. 1500). Guanshan lost one (of four) patch in 2022–2023 due to host tree death, accounting for population pattern, and in 2023–2024, the entire population was absent without any further host death. The Houbihu population was geographically limited but comparatively large in 2022–2023 (18 patches, 1108 individuals), but absent in 2023–2024. Both Lanyu populations increased substantively in the second year (2023–2024), driven by an increase in number of patches but comparatively smaller per‐patch population (ca. 52% of patches had five or fewer tubers).

**TABLE 1:** Population changes as measured by total number of tubers (bold; genetic individuals) and total patches (parentheses; spatially clustered tubers) for each year monitored.

Site	2021–2022		2022–2023		2023–2024	
	Tubers (patches)	%∆	Tubers (patches)	%∆	Tubers (patches)	%∆
Kenting	1501 (55)	—	2913 (209)	+94% (+280%)	1707 (138)	−41.5% (−34%)
Guanshan	155 (4)	—	75 (3)	−52% (−25%)	0 (0)	−100% (−100%)
Houbihu	N/A	—	1108 (18)	—	0 (0)	−100% (−100%)
Lanyu‐1	N/A	—	80 (5)	—	182 (21)	+128% (+320%)
Lanyu‐2	N/A	—	113 (11)	—	198 (33)	+75% (+200%)

*Note:* Percent changes (%∆) show net population change of the given year compared to the previous year for both tubers and patches; − and + indicate direction of change. N/A indicates that no population survey was conducted that year. Results show distinct and nonlinear year‐to‐year population fluctuations for all sites, with two sites (Houbihu and Guanshan) absent in the most recent survey. Surveys in Kenting were systematic transect surveys, while the remaining four sites had every tree base and surrounding area in the respective forest surveyed.

### 3.6 Reproductive Life History of Hosts

Host identification of a representative subset of hosts across all five populations (*n* = 81) suggests local host ranges are entirely composed of perennial woody species (78 trees, three lianas). Molecular analyses in Kenting identified the main host (43 of 48 samples) as *Diospyros maritima* (Ebenaceae), the dominant species in the 10‐ha plot and the surrounding uplifted karst forest ecosystem (> 50% of total stems; ca. 2100 stems/ha). The remaining identified hosts (one individual per species) were trees and lianas: tree species *Planchonella obovata* (Sapotaceae) and *Suregada aequorea* (Euphorbiaceae) are the 11th (40 stems/ha) and 21th (3 stems/ha) common woody species in the plot, respectively (Wu et al. 2011); liana species *Ticanto crista* (Fabaceae), *Hiptage benghalensis* (Malpighiaceae), and *Wisteriopsis reticulata* (Fabaceae) are the first (261 stems/ha), third (170 stems/ha), and fifth (40 stems/ha) most common lianas, and the third, fourth, and 12th most common woody species in the plot, respectively (Wu et al. 2007, 2011). Molecular analysis (*n* = 2) and root tracing (*n* = 2) in Guanshan identified *Macaranga tanarius* (Euphorbiaceae) as the only host. *M. tanarius* is a common but not dominant species in the Guanshan forest. In Houbihu, root tracing (*n* = 8) and proximity (*n* = 10) identified *Hibiscus taiwanensis* (Moraceae) as the only local host. In the Houbihu forest, *H. taiwanensis* is monodominant (> 90% of stems). In Lanyu‐1 and Lanyu‐2, molecular analysis (*n* = 21) identified *Suregada aequorea* (Euphorbiaceae; *n* = 16), *Tabernaemontana panacaqui* (Apocynaceae; *n* = 2), *Diospyros maritima* (Ebenaceae; *n* = 1), *Melicope triphylla* (Rutaceae; *n* = 1), and *Excoecaria cochinchinensis* (Euphorbiaceae; *n* = 1) as the local hosts. All species are common throughout the forests.

Host range does not reflect host availability at each site. Kenting host *D. maritima* is present in both Lanyu sites (one individual parasitized) and congenerics *D. philippensis* and *D. ferrea* are present but unparasitized in Guanshan; Lanyu host *S. aequorea* is present in Kenting (one individual parasitized) and Guanshan where is is unparasitized; Guanshan host *M. tanarius* is present but unparasitized in both Kenting (seventh most common species; 70 stems/ha) and Houbihu, and congeneric *M. sinensis* is a common species in both Lanyu sites.

### 3.7 Endophytic or Dormant Haustorial Parasite

**FIGURE 7:**
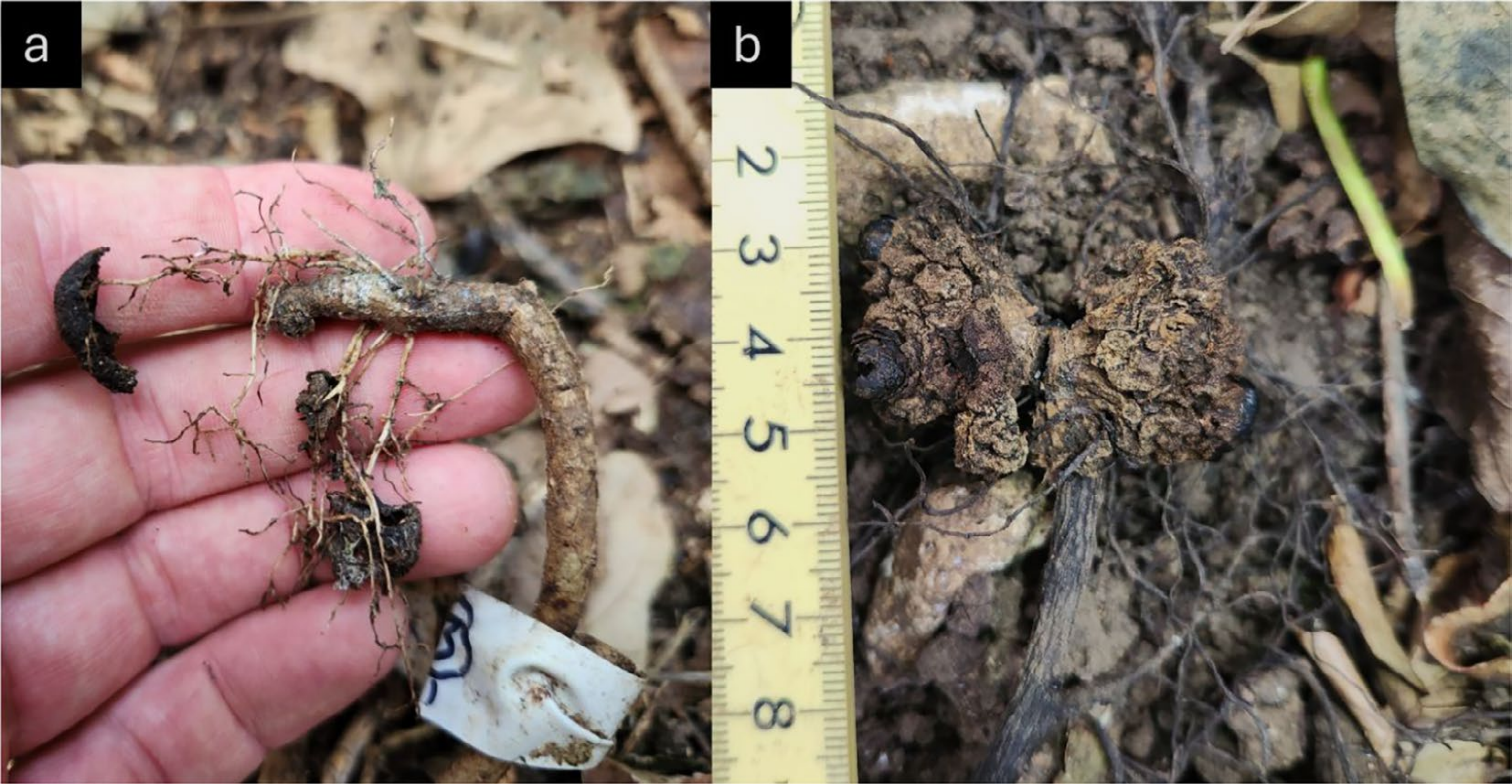
None of the 102 tuber‐root sections regrew a tuber in the following reproductive season. Once tubers deteriorate, the remaining root section remains healthy (a). In rare cases when the tuber is protected from rain, deterioration is slowed, and dried dead tubers persist on the host root (b).

No preselected root sections (*n* = 51) and cave protected dried tubers (*n* = 50) retained nor regrew a new tuber in the following season (Figure 7). Preselected roots were monitored throughout the vegetative growing season (Sep–Dec), while cave protected tubers were checked during and after peak reproductive season (December–March). All root sections at or upstream (tree side) of the point of tuber attachment the previous year remained living, though some root senescence was observed downstream of the point of tuber connection (*n* = 9). No galling was observed on any surveyed root (Dean 1954).

## 4 Discussion

### 4.1 Synthesis

We provide a consilience of evidence through a comprehensive literature review and six independent lines of inquiry, acquired from five geographically isolated populations variously over multiple reproductive cycles, to refute a perennial hypothesis and therefore describe *B. fungosa* ssp. *fungosa* in Taiwan to be an annual species. Our literature review found that no previous publication had investigated the question, and when designations of perenniality or annuality were made, they were inconsistent and without evidence or reference. Our field investigations thereafter provide clear refutation of perenniality or cryptic perenniality. Determining life‐history strategies of plants is not only vital to understanding their respective biology and ecology but also necessary to properly understanding their role within ecosystems. Our study provides the first quantified evaluation of the reproductive life‐history strategy of the obligate parasitic plant *B. fungosa* ssp. *fungosa* and provides a replicable methodology for future work to quantitatively determine annuality or perenniality—including plasticity—of the important, but understudied, cohort of parasitic plants.

### 4.2 Literature Review

Our literature review provided the first comprehensive inquiry into the published record of reproductive life‐history strategies within the genus *Balanophora*. While most publications did not report a strategy (ca. 87%), publications variously reported perenniality, annuality, bienniality, or both. Given the absence of any primary study investigating perenniality or annuality, it is probable that these designations were based on inferences from short‐term field observations or assumptive determinations passed on through time, and not long‐term studies. Our results show that while patches do change in size year‐to‐year, and come and go over years, they are largely static in spatial distribution. Furthermore, most seed dispersal appears to be through gravity (Padgett et al., *unpublished data*), meaning that once a patch is established, it is highly likely to remain over years via abundant seed source and availability of host; it is plausible that researchers seeing a recurring patch of flowering *Balanophora* assumed it was the same individuals re‐flowering (perenniality) rather than new individuals being recruited each year (annuality). The complex life cycle and cryptic (typically subterranean) habits of *Balanophora* could lead to misinterpretations during short‐term and/or observational data.

The presence of contradicting determinations within the literature is in no way evidence of error; congeneric and conspecific variation in life‐history strategies is not uncommon (Boyko et al. 2023; Felmy et al. 2022; Kelly et al. 2021), including reversions to ancestral perenniality from a derived annuality (Monroe et al. 2019; Verma and Kaul 2022). However, the lack of any primary investigation into the question does suggest a substantial gap in our understanding of the species. To effectively address the issues of determination and plasticity, we recommend direct quantitative investigations of parasitic plant strategies—including other *Balanophora* species—to enhance our understanding of these unique and important plants.

### 4.3 Cryptic Perenniality

Cryptic perenniality in parasitic plants could occur via host range life history (perennial parasite occupying annual host species), host range population dynamics (host species undergoing high mortality), or the parasite exhibiting endophytic/dormant strategies. Host ranges were found to be perennial trees (*n* = 76; ca. 96%) or lianas (*n* = 3; ca. 4%) that have not undergone substantial long‐term mortality (Wu et al., *unpublished data*) nor disturbance (i.e., typhoon) related mortality (Wu & Padgett, *unpublished data*) that could explain parasite population changes. Endophytism and dormancy have been reported in parasitic plants (Těšitel 2016; Kelly and Denhof 2022), including Balanophoraceae (Pellissari et al. 2022), but were not found in our study. Together, these data refute the hypothesis of cryptic perenniality and instead provide support for annuality.

### 4.4 Reproductive Life‐History Plasticity

Annuality has independently evolved from the ancestral perenniality thousands of times among flowering plants (Friedman 2020) and the resulting dispersion of each strategy among lineages shows minimal phylogenetic pattern, making relatedness a poor indicator (Böhle, Hilger, and Martin 1996; Heidel et al. 2016; Van Kleunen 2007). While there is evidence of environmental patterns at local (Gould, Chen, and Lowry 2017; Lowry et al. 2019; Lundgren and Des Marais 2020; Stearns 1992) and global scales (Poppenwimer, Mayrose, and DeMalach 2023), geographically proximate congenerics and conspecifics have been shown to exhibit different strategies, as well as some annual species reverting to ancestral perenniality—such as insular *Echium* spp. (Boraginaceae; Böhle, Hilger, and Martin 1996), *Nemesia* spp. (Scrophulariaceae; Datson, Murray, and Steiner 2008), and *Anisomeles indica* (Lamiaceae; Verma and Kaul 2022)—while closely related sympatric congenerics remained perennial (e.g., Heidel et al. 2016; Hjertaas et al. 2023; Vico et al. 2016). This phylogenetic complexity and population‐level plasticity of flowering plant reproductive strategies making it difficult to determine the strategy of a species without primary investigation of the local population (Hall and Willis 2005; Van Kleunen 2007). For parasitic plants, the potential for plasticity and population‐level difference in strategy is further driven by differences in host species, known to be an evolutionary driver (e.g., de Vega et al. 2024). To define a strategy for a species—particularly cryptic herbaceous species—it is necessary to test the question over a representative sample across a wide geographical range.

Our methodology and results provide evidence of annuality and a lack of plasticity within any of the five geographically isolated populations with unique habitat conditions and local host ranges. The sites on Taiwan proper (*n* = 3) are found on uplifted coral reef limestone, have very shallow and nutrient poor alkaline soils, and have distinct dry season during which time no rain falls (October–May) followed by an intense wet season (June–September) (Wu et al. 2011). Lanyu is a volcanic island, and sites (*n* = 2) are underlain by higher nutrient acidic soils of andesite origin and experience year‐long precipitation. Further habitat diversity exists between sites relating to proximity to ocean (salt intrusion and sea spray), topography, canopy cover, soil moisture and pH, climate, and elevation (ca. 2 m—300 m a.s.l.). Given this among‐site diversity, we would expect a signal of plasticity if it was present within the populations, especially with such site‐specific host ranges that could have profound impacts through direct host–parasite “molecular dialog” (e.g., Clarke et al. 2019; Troncoso et al. 2010). While it is not plausible to make a conclusion of the larger range of the species, this study does provide a basis to consider that *B. fungosa* ssp. *fungosa*, and potentially the *B. fungosa* complex (*B. f*. ssp. *fungosa, B. f*. ssp. *indica, B. f*. var. *globosa*), may be an annual throughout its entire global range.

### 4.5 Ecological, Evolutionary, and Conservation Implications

Determining the reproductive life‐history strategy of a species is more than documentation of its natural history. Due to the distinct differences in the biology, ecology, and population genetics of perennial and annual species, a quantitative assessment of the strategies is essential for data interpretation and effective conservation management (e.g., Willis et al. 2007). Interpreting data from an annual species through a perennial lens will have potentially significant implications, wherein inaccurate or incorrect conclusions are drawn. Population genetic implications include, but are not limited to, interpretations of gene flow, selection, bottlenecks, and speciation dynamics (Gaut, Díez, and Morrell 2015); differences in drivers of genetic structure between conspecific populations (Austerlitz et al. 2000; Hamrick and Godt 1996; Loveless and Hamrick 1984); expectations of genetic diversity and resilience (Hwang et al. 2019); or implications of effective population sizes (Espeland and Rice 2010; Nunney 2002). Perennial and annual species also differ in phenological and reproductive responses to environmental cues presented by cyclic (Pagel et al. 2008), chronic (Monroe et al. 2019; Reed et al. 2021), and stochastic disturbances (Rehman, Bahadur, and Xia 2023; Tan and Swain 2006), and have unique drivers of and expectations for population dynamics (Edelfeldt, Bengtsson, and Dahlgren 2019; Stouffer 2022), germination ecologies (Rees and Long 1992; Ten Brink et al. 2023), competition dynamics (Crawley and May 1987; Soppe, Viñegra De La Torre, and Albani 2021), and potential responses to climate change (Compagnoni et al. 2021; Morris et al. 2008). Incorrectly assigning perenniality to an annual species can therefore incur substantial to significant errors in analyses and interpretation of data, thereby permitting researchers to make incorrect conclusions and thereafter inappropriate conservation management decisions.

Specific to parasitic plants, the perenniality or annuality of a species will infer different impacts on its hosts (Press and Phoenix 2005), though structured investigation on comparative impacts remains lacking. In theory, perennial parasitic species will have a more substantive impact on a given individual, while annual parasitic species have their impacts more dispersed across the population. Perennial will infect a given host(s) individual and from it remove resources over its lifetime entire lifetime. This long‐term infection can potentially have important impacts on host growth, reproduction, and mortality (Bell and Adams 2011; Matthies 2017; Press and Phoenix 2005; Těšitel et al. 2021; Watling and Press 2001), thereby affecting individual host dynamics within a forest (e.g., Moncalvillo and Matthies 2023). Restarting their population each year from new and soil banked seeds, annual species have a greater potential to switch host individuals or species with each generation (year), making the net impact on any given individual comparatively minimal and analogous to a random disturbance event rather than a lifelong affliction.

These differences also manifest when studying host–parasite co‐evolution and speciation of conspecific parasite populations. Given their direct and requisite connection to a host, the host minimizes potential environmental influence and instead the speciation and expression of plasticity observed among parasitic plants is substantially—and differentially—impacted by host species identity (Brown and Tellier 2011; Clarke et al. 2019; de Vega et al. 2024; Irving and Cameron 2009; Tellier and Brown 2011). Beyond the water and nutrients that are obtained by the parasite from the host, there is constant—often bilateral—exchange of proteins (Liu et al. 2020), mRNAs and RNAs (Kim et al. 2014; LeBlanc et al. 2013; Westwood and Kim 2017), and genes that have been found to be functionally integrated into parasite genomes (Davis and Xi 2015; Richardson and Palmer 2006; Xi et al. 2013). Assuming perenniality—long‐term connection and stronger co‐evolution and site‐specific speciation potential through continued association with a single host—in an annual species that is showing slower than expected divergence can lead researchers to under‐assign evolvability or co‐speciation potential.

### 4.6 The Case for Annuality

Our study provides a strong consilience of evidence supporting *B. fungosa* ssp. *fungosa* as an annual species in Taiwan. We tested the hypothesis of perenniality and cryptic perenniality as this is expected to be the ancestral state within flowering plants (Friedman 2020; Hjertaas et al. 2023; Soltis et al. 2013; Stebbins 1950). However, there currently exists no determination as to whether the ancestral lineage of either *Balanophora* or Balanophoraceae itself maintained the ancestral state or had already derived annuality. We found no ambiguity in our results and therefore conclude that *B. fungosa* ssp. *fungosa* is an annual species in Taiwan: 0% survived post‐reproduction, 0% were present between reproductive seasons, tuber number/volume growth clearly showed increases over time starting from zero each year, year‐to‐year population fluctuation was substantial, 100% of identified hosts were perennial, and 0% of root sections showed signs of endophytic or dormant tubers.

**FIGURE 8:**
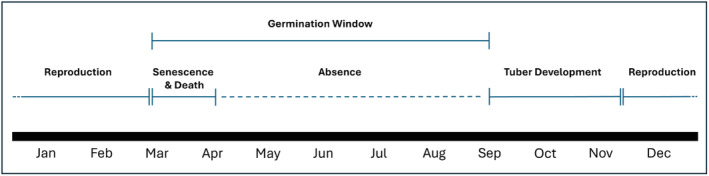
Schematic diagram of the life of a *B. fungosa* ssp. *fungosa* individual. Tubers first become noticeable (< 1 mm length) in late September at the earliest, vegetatively growing until reproductive (flowering and fruiting) late November to early March. After reproduction tubers are absent in the forest. Germination requirements and period remains unknown, represented here as a window and not a specific time.

In all five sites, our results support post‐reproductive senescence then death of the entire population (sensu Thomas 2013) followed by the recruitment of entirely new individuals for the following years population. *B. fungosa* ssp. *fungosa* undergoes a single coordinated reproductive event and after fruiting individuals senesce and die, leaving them absent from the respective sites in the spring and summer. New small, sub‐millimeter diameter tubers develop by the early fall, undergoing vegetative development until they reach maturity and develop inflorescence‐analogous reproductive structures in the winter. Following reproductive maturity and fruiting, all inflorescence‐analogous reproductive structures begin to senesce, eventually falling to the soil surface and tubers begin to die, completing a single life cycle (Figure 8).

Beyond our focal species, we encourage researchers studying plants to increase the quantitative determination of reproductive life‐history strategies, particularly for cryptic and understudied taxa. This will improve the accuracy of our knowledge and understanding of the ecology, evolution, and conservation of both species and ecosystems.

## Author Contributions

**Trevor Padgett:** conceptualization (equal), data curation (lead), formal analysis (equal), methodology (equal), visualization (equal), writing – original draft (lead), writing – review and editing (equal). **Huei‐Jiun Su:** conceptualization (equal), funding acquisition (supporting), methodology (equal), resources (supporting), supervision (equal), writing – original draft (supporting), writing – review and editing (equal). **Shu‐Hui Wu:** data curation (equal). **Li‐yen Huang:** data curation (equal). **Yiching Lin:** conceptualization (equal), formal analysis (equal), funding acquisition (equal), methodology (equal), resources (equal), supervision (equal), writing – original draft (equal), writing – review and editing (equal).

## Conflicts of Interest

The authors declare no conflicts of interest.

## Supporting information


**Data S1.** Supporting Information.

## Data Availability

Data are available at https://github.com/trevor‐padgett/Balanophora‐Annual‐Data.
