# Supplementary material for: Quantitative Approach for Determining Reproductive Life‐History Strategies of Parasitic Plants: A Case Study in Balanophora
Source: Ecol Evol. 2025 Jan 11;15(1):e70746. doi: 10.1002/ece3.70746 (PMC11724198; doi:10.1002/ece3.70746)
Supplement: Supplementary file 1 — Data S1. Supporting Information. [file ECE3-15-e70746-s001.docx]

| **Reported Strategy** | **Publication Type** | **Title** | **Year** | **Author(s)** |
| --- | --- | --- | --- | --- |
| Annual | Primary | 月本屋つちとりもち科植物ノ形態学的並二 生態学的研究 | 1942 | Watanabe (1942) |
| Biennial or Perennial | Herbarium | Plants of the World Online | 2023 | Kew Gardens (accessed Aug 2024) |
| Perennial & Annual | Description | The Families of Flowering Plants 3rd Ed. | 1973 | Hutchinson (1973) |
| Perennial & Annual | Flora | Flora of Shenzen Vol. II | 2010 | Li (ed.) (2010) |
| Perennial | Flora | Flora Hainanica Vol. III (1965) | 1965 | Chun & Chang (1965) |
| Perennial | Flora | Revised Handbook to Flora of Ceylon | 1990 | Dassanayake & Fosberg (1990) |
| Perennial | Flora | Flora of Hong Kong | 2008 | Hong Kong Herbarium & South China Botanical Garden (2008) |
| Perennial | Primary | Floral Biology and pollination in Balanophoraceae | 2002 | Kawakito & Kato (2002) |
| Perennial | Primary | Polysaccharides from Balanophora harlandii Hook: Isolation, 2 Characterization, and Anti-Inflammation Activities | 2023 | Li et al (2023) |
| Perennial | Floristic Survey | 土名對照滿鮮植物字彙 | 1934 | Murata (1934) |
| Perennial | Flora | Flora Fujianica | 1991 | Provincial Science & Technology Commission Writing Team (1991) |
| Perennial | Floristic Checklist | Wild Flowers of Japan: Herbaceous Plants (Vol II) | 1981 | Satake et al. (1981) |
| Perennial | Flora | Flora of Guam | 1970 | Stone (1970) |
| Perennial | Flora | Biodiversity of Lao PDR | 2017 | Woo-Shin et al. (2017) |
| Perennial | Primary | Two new species of Balanophora (Balanophoraceae) from Southern China | 2021 | Yu et al. (2021) |
| Perennial | Flora | Flora of Hunan Hupingshan Vol. II | 2008 | Zhang & Zhang (Eds.) (2008) |
| None | New Record | *Balanophora papuana* (Balanophoraceae), a newly recorded species for Thailand | 2024 | Sakdapipanich et al. (2024) |
| None | Primary | Host and anatomy of Balanophora fungosa var. indica | 2017 | Alex & Vidyasagaran (2017) |
| None | Flora | Flora of New Zealand (1982) | 1982 | Allan & Hasselberg (1982) |
| None | Floral Checklist | Inventory of parasitic plants in the natural tourist park around lake sicikeh-cikeh, lae hole village, parbuluan district, dairi regency, north sumatra. | 2020 | Azizah (2020) |
| None | Flora | Flora of Java (Vol II-1) | 1965 | Backer & van Den Brink (1965) |
| None | Endangered Species | Vietnam Red Book | 2007 | Ban et al (2007) |
| None | Review | The genus *Balanophora* (Balanophoraceae) in Sabah, Malaysia | 2003 | Barkman et al. (2003) |
| None | Primary | Plastomes in the holoparasitic family Balanophoraceae | 2021 | Ceriotte et al (2021) |
| None | Primary | Balanophora subcupularis (Balanophoraceae), a new record from Thailand | 2022 | Chaiwerawattana et al. (2022) |
| None | Primary | The embryology of Balanophora | 1914 | Chamberlain (1914) |
| None | Primary | Balanophora genomes display massively convergent evolution with | 2023 | Chen et al. (2023) |
| None | Primary | A Preliminary Report of Small Mammal Frugivory on B. harlandii | 2012 | Conran & Li (2012) |
| None | Flora | Flora of Bombay | 1958 | Cooke (1958) |
| None | Flora | Flora of Kwangtung & Hong Kong | 1912 | Dunn & Tutcher (1912) |
| None | Primary | Development and morphology of Balanophora flowers | 2009 | Eberwein et al. (2009) |
| None | Review | Contributions to our kowledge of Balanophora | 1935 | Ekambaram & Panje (1935) |
| None | Primary | Blüte und Blütenstand der Gattung *Balanophora* | 1945 | Fagerlind (1945) |
| None | Review | On new species of *Balanophora* and *Thonningia* | 1886 | Fawcette (1886) |
| None | Flora | Flora of Australia | 2023 | Flora of Australia Online (Accessed September 2024) |
| None | Flora Checklist | The Forster Pacific islands collections from Captain Cook’s Resolution Voyage | 1993 | Fosberg (1993) |
| None | Primary | An ultrastructural study of the tuber strands of Balanophora (Balanophoraceae | 1990 | Gedalovich-Shedletzky & Kujit (1990) |
| None | Key | Thonners Analytical Key to the Families of Flowering Plants (1981) | 1981 | Geesink et al. (1981) |
| None | Primary | On Occurrence of Three Parasitic Angiosperms within Neora Valley National Park | 2021 | Ghosh et al (2021) |
| None | Primary | The Pollination Mechanism in Balanophora abbreviate Blume | 1975 | Govindappa & Shivamurthy (1975) |
| None | Primary | On the Indian Species of Balanophora, and on a new Genus of the Family Balanophoraceae. | 1844 | Griffith (1844) |
| None | Review | Balanophora Studien (German) | 1945 | Guttenberg (1945) |
| None | Primary | Illustration of two rare Balanophoraceae of equatorial Africa belonging to the genera *Chlamydophytum* Mildbr. and *Balanophora* Forst. | 1978 | Halle (1978) |
| None | Primary | A new species of Balanophora from the Malay Peninsula | 1980 | Hambali (1980) |
| None | Floral Checklist | A Catalogue of the Herbarium Specimens from Captain Cook's First and Second Expeditions Housed in the Copenhagen Herbarium | 1998 | Hansen & Wagner (1998) |
| None | Flora | The Genus Balanophora: A Taxonomic Monograph | 1972 | Hansen (1972) |
| None | Family Description | A monographs of the neotropical Balanophoraceae | 1980 | Hansen (1980) |
| None | Flora | Balanophoraceae of the Pacific | 1982 | Hansen (1982) |
| None | Review | Balanophora species published 1971 - 1998, mostly hm China and Japan | 1999 | Hansen (1999) |
| None | Floral Checklist | Flora Montana Formosae | 1908 | Hayata (1908) |
| None | Primary | Contributions to our knowledge of the genus Balanophora (Translated) | 1907 | Heinricher (1907) |
| None | Primary | Van Tieghem's Anschauungen über den Bau der Balanophora-Knolle | 1908 | Heinricher (1908) |
| None | Flora | Flowering Plants of the World (1978) | 1978 | Heywood (1978) |
| None | Species Description | On a New Genus of Balanophoraceae from New Zealand and Two New Species of Balanophora | 1859 | Hooker (1859) |
| None | Primary | Composite bundles at host parasite interface in Balanophora | 1995 | Hsiao et al. (1995) |
| None | Primary | Genetic diversity of Balanophora fungosa and its conservation in Taiwan | 2010 | Hsiao et al. (2010) |
| None | Flora | Flora Malesiana Online | 2023 | https://floramalesiana.org/new/ |
| None | Flora | PNGplants Database Online | 2023 | https://www.pngplants.org/PNGdatabase.html |
| None | Flora | Flora of Taiwan | 1984 | Huang (1984) |
| None | Primary | Endophytic fungi associated with a holoparasitic plants Balanophora japonica | 2016 | Ikeda et al. (2016) |
| None | Plant List | Orders and Families of Seed Plants of China | 1993 | Keng et al (1993) |
| None | Primary | Identifying host species of Balanophora fungosa indica | 2013 | Kim & Won (2013) |
| None | Primary | Extreme plastomes in Balanophora are not the norm | 2023 | Kim et al. (2023) |
| None | Primary | Surface features of the leaves of BManophoraceaeA family without stomata | 1990 | Kujit & Dong (1990) |
| None | Family Description | The Families and Genera of Vascular Plants Vol XII: Satalales and Balanophorales | 2015 | Kujit & Hansen (2015) |
| None | Primary | A new species of balanophora from hong kong | 2003 | Lau et al. (2003) |
| None | Floral Checklist | Composition, Origin, and Affinities of the Madagascan Vascular Flora | 1978 | Leroy (1978) |
| None | Primary | Balanophora aphylla (Balanophoraceae), a new holoparasitic species | 2019 | Luu et al. (2019) |
| None | Primary | Observations on the flora of Japan | 1909 | Makino (1909) |
| None | Flora | Flora of China (eFlora) | 2008 | Missouri Botanical Garden (2008) |
| None | Primary | Morphology and distribution of Balanophora fungosa | 1988 | Murata (1988) |
| None | Plant ID | Handbook to Reference and Identification | 1997 | Nguyen (1997) |
| None | Primary | First record of Balanophora tobiracola Makino (Balanophoraceae) from Viet Nam | 2018 | Nguyen et al. (2018) |
| None | Primary | Botanical Characteristics of Balanophora (Balanophora) | 2012 | Nguyen et al. (2021) |
| None | Flora | Flora of Japan (English Version) | 1984 | Ohwi, Meyuer, & Walker (Eds.) (1984) |
| None | Flora | A Pocket Flora of the Sirumalai Hills, South India | 2001 | Pallithanam (2001) |
| None | Primary | Balanophora coralliformis (Balanophoraceae), a new species from Mt. Mingan, Luzon, Philippines | 2014 | Pelser et al. (2014) |
| None | Primary | Musky rat Kangaroo feed from male flowers | 2017 | Pierce & Olga (2017) |
| None | Primary | A note on the distribution of Balanophora polyandra Griff. (Balanophoraceae) in and around Neora Valley National Park, West Bengal, India | 2020 | Rai et al. (2020) |
| None | Primary | Occurrence of parasitic Balanophora in India | 2015 | Rao et al. (2015) |
| None | Primary | The parasitic interface between Balanophora indica and Cynometra ramiflora | 1985 | Russo et al. (1985) |
| None | Primary | Establishment structure and morphology of the tuber of Balanophora | 1981 | Shivamurthy et al. (1981a) |
| None | Primary | Ontogeny and Organization of the Inflorescence in Balanophora | 1981 | Shivamurthy et al. (1981b) |
| None | Plant List | Seed Plants of the Karst Region of China Vol. I | 2006 | Shui et al. (2006) |
| None | Flora | Flora Vitiensis | 1985 | Smith (1985) |
| None | Flora | Flora of Thailand | 1972 | Smitinand & Larsen (eds.) (1972) |
| None | Flora | Concise Flowers of the Himalayas | 1988 | Stainton & Farrer (1988) |
| None | Primary | Morphology and phylogenetics of two holoparasitic plants (Balanophora) and their hosts | 2012 | Su et al. (2012) |
| None | Primary | Novel genetics and reduced plastid in Balanophoraceae | 2019 | Su et al. (2019) |
| None | Primary | Flower visitors to Balanophora fungosa var indica | 2014 | Suetsugu & Aoyama (2014) |
| None | Primary | A non-photosynthetic plant provides the endangered amani rabbit with vegetative tissues as a reward for seed dispersal | 2023 | Suetsugu & Hashiwaki (2023) |
| None | Primary | Brood site pollination mutualism | 2020 | Suetsugu & Hisamatsu (2020) |
| None | Primary | The cockroach - a largely overlooked pollinator | 2022 | Suetsugu & Yamashita (2022) |
| None | Primary | Infestation of the non-photosynthetic plant Mitrastemon yamamotoi | 2019 | Suetsugu & Yoshiyasu (2019) |
| None | Primary | Avian seed dispersal of Balanophora yakushimensis | 2020 | Suetsugu (2020) |
| None | Thesis | Flora Taman Nasio Gede Pangrango | 1992 | Sunaryo (1992) |
| None | Species Description | Fruit/seed morphology of Balanophora fungosa J.R. & G. Forst. ssp. indica (Arn.) B. Hansen var. globosa (Jungh.) B. Hansen (Translated) | 1999 | Sunaryo (1999) |
| None | Primary | Spissoimyces endophytica a new endophytic fungus from Thailand | 2018 | Suwannarach et al. (2018) |
| None | Review | Parasitic plant cultivation: examples, lessons learned and future directions | 2022 | Thorogood et al (2022) |
| None | Primary | Balanophora dioica - a new record for south India from Western Ghats | 2016 | Thriveni et al. (2016) |
| None | Primary | Note on Balanophora Thwaitesii | 1886 | Trimen (1886) |
| None | Primary | Balanophora subcupularis P C Tam (Balanophoraceae) - New Record | 2017 | Tung et al (2017) |
| None | Flora | Flora Malesiana Online | 2023 | Unknown Citation |
| None | Flora | Melesian Seed Plants | 2001 | van Balgooy (2001) |
| None | Flora | Flora of Bashahr Himalayas | 1977 | Varghese (ed) (1977) |
| None | Primary | Pollination Ecology of Balanophora | 2018 | Visolobokov & Galinskaya (2018) |
| None | Floral Checklist | Vascular Plants of the Hengduan Mountains Vol. I | 1993 | Wang et al. (1993) |
| None | Primary | Phytochemicals and biological studies of plants from the genus Balanophora | 2012 | Wang et al. (2012) |
| None | Primary | Entwicklung der Knollen von Balanophora nipponica | 1936 | Wantanabe (1936) |
| None | Species Description | Uber die Verbreitung and die Lebensweise von Balanophora nipponica 1 | 1935 | Watanabe (1935) |
| None | Primary | Diversity and communities of culturable endophytic fungi in Balanophora polyandra | 2022 | Wu et al. (2022) |
| None | Plant ID | Wild Plants of Shenzen, China | 2000 | Xing & Yu (eds.) (2000) |
| None | Primary | Association of Balanophora elongata Blume with understory plants in the Cibodas Royal Garden Area Forest (Translated) | 2017 | Zuhri (2017) |
| none | Primary | First cavernicolous record of the holoparasitic *Balanophora abbreviata* Blume (Balanophoraceae), from Tayabas, Quezon, Philippines | 2024 | Lambio et al (2024) |
| none | Primary | Balanophora papuana Schltr. (Balanophoraceae), a Neglected Holoparasite Species: Rediscovery for Indonesia | 2019 | Damayanto & Riastiwi (2019) |
| none | flora | The flora of the Bokor Plateau, southeastern Cambodia: a homage to Pauline Dy Phon | 2017 | Rundel & Middleton (2017) |
| none | Review | Phytographic review of Vietnam and adjecent areas of eastern indochina | 2003 | Averyanov et al (2003) |
| none | Review | Some Endangered Plants and Threatened Habitats in South East Asia | 1969 | Qureshi & Kaul (1969) |
| none | Primary | The systematic significance of surface features of the *Balanophora* tuber (*Balanophoraceae*) | 1990 | Kujit & Dong (1990) |
| None | New Record | Balanophora papuana (Balanophoraceae), a newly recorded species for Thailand | 2024 | Sakdapipanich et al. (2024) |
| none | Checklist | Checklist of Parasitic Plants in Marilog District, Southern Philippines | 2024 | Tubongbanua et al. (2024) |
| none | Review | Balanophora-Studien | 1945 | von Guttenburg (1945) |
| none | New Record | New Species on Genus Balanophora (Latin) | 1983 | Chang & Tam (1983) |

**S1:** Summary table of retrieved documents that were included in literature review. In total 122 ecology-based publications were included; publications relating to pharmacological, medicinal, or cultural aspects were not included as description of life history strategies would not be an expected component. Literature list includes search results obtained from Google Scholar, Google search, JSTOR, herbaria (online and physical), and herbarium library at Academia Sinica, Taiwan. Determinations were either absent (87%) or reported as perennial (9%), annual (1%). Biennial/multi-year (1%), or as both perennial and/or annual (2%). No publication investigated the life history strategy of *Balanophora* directly, and no reported strategy was supported by reference or data. *Reported Strategy* reports the reproductive life history strategies that were described in each publication. *Publication Type* reports the type of publication the determination came from; for herbarium samples (online or physical) a one determination was made for the entire collection of *Balanophora*. *Title* and *Year* report the publication data. *Author(s)* reports the simplified author citation.
